# Effect of Hepatic Macrophage Polarization and Apoptosis on Liver Ischemia and Reperfusion Injury During Liver Transplantation

**DOI:** 10.3389/fimmu.2020.01193

**Published:** 2020-06-26

**Authors:** Liping Ye, Saiqin He, Xinli Mao, Yu Zhang, Yue Cai, Shaowei Li

**Affiliations:** ^1^Department of Gastroenterology, Taizhou Hospital of Zhejiang Province Affiliated to Wenzhou Medical University, Linhai, China; ^2^Endoscopy Center, Taizhou Hospital of Zhejiang Province Affiliated to Wenzhou Medical University, Linhai, China

**Keywords:** liver transplantation, kupffer cells, ischemia reperfusion, apoptosis, polarization

## Abstract

Ischemia-reperfusion (I/R) injury is injury caused by a limited blood supply and subsequent blood supply recovery during liver transplantation. Serious ischemia-reperfusion injury is the main cause of transplant failure. Hepatic I/R is characterized by tissue hypoxia due to a limited blood supply and reperfusion inducing oxidative stress and an immune response. Studies have confirmed that Kupffer cells (KCs), resident macrophages in the liver, play a key role in aseptic inflammation induced by I/R. In liver macrophage polarization, M1 macrophages activated by interferon-γ (IFN-γ) and lipopolysaccharide (LPS) exert a pro-inflammatory effect and release a variety of inflammatory cytokines. M2 macrophages activated by IL-4 have an anti-inflammatory response. M1-type KCs are the dominant players in I/R as they secrete various pro-inflammatory cytokines that exacerbate the injury and recruit other types of immune cells via the circulation. In contrast, M2-type KCs can ameliorate I/R through unregulated anti-inflammatory factors. A new notion has been proposed that KC apoptosis may influence I/R in yet another manner as well. Management of KCs is expected to help improve I/R. This review summarizes the effects of hepatic macrophage polarization and apoptosis on liver I/R.

## Introduction

Advances in surgical procedures and the application of immunosuppressive technologies have made liver transplantation (LT) the optimal treatment for almost all types of end-stage liver disease (1). The current technology has brought the 1-year survival rate of patients receiving LT to more than 80%, but some problems associated with LT must still be addressed.

The most important factor is I/R (2), which includes ischemic liver damage and subsequent reperfusion injury. It is a two-stage pathophysiological process that occurs during liver resection and liver transplantation (3). Hepatic ischemic injury is characterized by ATP and glycogen depletion as well as cellular metabolic stress caused by mitochondrial dysfunction, all of which lead to initial cell death (4). Subsequent reperfusion injury refers to the phenomenon wherein the liver sustains severe damage after the blood flow and reoxygenation are restored. During this process, metabolic disorders and a large number of reactive oxygen species (ROS) and cytokines or chemokines stimulate various immune cells to produce a severe inflammatory response (5). Multiple studies have shown that I/R damage to the liver tissue involves a series of pathological processes (6–8). I/R-related macrophage activation and related inflammatory factor explosions are key to graft dysfunction and even the occurrence of primary non-function (9, 10).

The liver has several types of innate immune cells, including the inherent macrophages known as Kupffer cells (KCs) (11), dendritic cells (DCs), and natural killer T (NKT) cells. KCs are an important part of the innate immune response and the largest fixed macrophage population in the body, accounting for 40–65% of the total liver non-parenchymal cells (12). Polarization and apoptosis of KCs have been recognized as important topics concerning hepatic I/R injury.

We herein review the dual role of KC polarization and apoptosis in I/R.

## Quiescence and Activation of KCs in I/R

KCs are liver macrophages located in the liver sinusoid and play a key role in the immune response. Under steady-state conditions, KCs have close contact with circulating blood flowing from the portal vein or hepatic artery, which allows them to devour most pathogens. In addition, they are responsible for removing other substances, including cell debris and immune complexes (13). It is now believed that KCs in healthy livers exhibit a “tolerogenic” phenotype that can maintain immune tolerance. However, in a disease state, such as IR, a phenotypic change occurs, which is involved in the immune response (14, 15). The activated KCs secrete pro-inflammatory cytokines and induce a subsequent inflammatory response. The activated KC function is, thus, enhanced, and the cells produce a number of different cytokines and chemokines, such as IL-6, TNFα, Nos2, Arg1, and Mrc1 (16).

## Polarization of KCs in I/R

KCs are usually polarized to the M1 type during hepatic IR damage (17). It is important that macrophage polarization be understood as a spectrum of transformation. There is no pure M1- or M2-type macrophage population, and these phenotypes undergo transformation according to the stimulation signals they receive (18). M2 macrophages can counteract the pro-inflammatory effects of M1 macrophages during the process of inhibiting pro-inflammatory signaling (19). The pathogenesis of type 2 diabetes (T2D) is chronic hyperinsulinemia caused by systemic and hepatic insulin resistance (IR). Without intervention, pancreatic β-cell failure will result. IR and T2D are commonly observed in individuals with non-alcoholic fatty liver disease (NAFLD) (20).

Hyperglycemia reduces the secretion of IL-10 by inducing the reduction of Arg1 and Mrc1 expression and the activation of the STAT3 and STAT6 signaling pathways to inhibit M2-like KC polarization. It was demonstrated that hyperglycemia induces high inflammatory activation of KCs during liver I/R. Therefore, the hyperglycemia-induced overexpression of C/EBP homologous protein (CHOP) inhibits the polarization of M2-like KCs secreted IL-10, leading to inflammatory activation of KC during liver I/R (21).

PPAR-γ exerts a protective effect by inducing KCs to polarize to M2-type macrophages (22). Sphingosine-1-phosphate (S1P) and sphingosine-1-phosphate receptors (S1PRs) are involved in metabolic and inflammatory diseases. Hyperglycemia exacerbates I/R by promoting M1 polarization and inhibiting M2 polarization, specifically triggering S1P/S1PR3 signaling (23).

Min et al. used myeloid-specific HO-1 gene knockout (mHO-1-KO) and transgenic (mHO-1-Tg) mice to delete or overexpress HO-1, verifying that myeloid HO-1 expression improves liver IR damage by promoting macrophage M2 phenotypic polarization. Interestingly, in human liver transplantation biopsies, subjects with higher HO-1 levels showed a lower expression of M1 markers and higher expression of M2 markers as well as reduced hepatic damage and an improved prognosis (24). Soluble fibrinogen-like protein 2 (sFGL2) promotes the secretion of anti-inflammatory cytokines (IL-10, TGF-β) and the high expression of CD206 and inhibits the activity of STAT1 and NF-κB signaling pathways. sFGL2 improves the prognosis of LT by inducing KC M2 polarization in rat orthotropic liver transplantation (OLT) models (25).

## Apoptosis of KCs and I/R

A transplanted liver is not only directly affected by I/R but also damaged by apoptosis during transplantation (26). At present, inhibiting and regulating the KC function has become a hot topic in protecting transplanted livers from I/R. GdCl3 induces KC apoptosis and reduces IR damage in liver transplantation (27). In the early stages of I/R, the activation of KCs and overexpression of inflammatory factors, such as TNF-α, are the main causes of graft dysfunction after transplantation (28). KC activation inhibitors are widely used drugs that reduce liver damage in donor animals (29).

KCs exert a protective effect on liver tissue I/R during transplantation (30). Upregulating the expression of IL-10 can protect against I/R in steatotic liver, and more importantly, KC still has a hepatoprotective effect in steatotic liver (31).

Mesenchymal stem cells (MSCs) have been proposed as promising treatments for certain liver diseases, and studies have found that MSCs also have a protective role in “donated after circulatory death” LT. In a mouse non-heartbeat LT model, the survival rate and cytokine, and chemokine expression of animals with and without MSC infusion were compared. It was found that the protective effect of MSCs on I/R was caused by the secretion of PGE2, which regulated the TLR4-ERK1/2-caspase3 pathway and inhibited KC apoptosis (32). KCs, as an important part of the reticuloendothelial system, are responsible for the clearance and detoxification of intestinal Lipopolysaccharide (LPS) (33). Under conditions of KC depletion, LPS is not effectively metabolized in the liver and may continue to cause damage (34). However, indiscriminately reducing KC activation is not an effective way to reduce I/R in steatosis liver (31).

## Crosstalk of Apoptosis and Polarization of KCs in I/R

IL-10 is a key anti-inflammatory cytokine produced by immune cells when activated (35). The macrophage phenotype can be reprogrammed from M1 to M2 by upregulating endogenous IL-10 (36). M1 polarization is promoted while M2 polarization is inhibited to specifically aggravate liver I/R (23). In the absence of IL-10 signaling, mTOR can promote the accumulation of damaged mitochondria in macrophages, leading to the dysregulation of NLRP3 inflammatory bodies and production of excess IL-1β (37). In the polarization of primary human macrophages, the expression of apoptosis inhibitor (IAP) protein is different in macrophages with different polarizations. NLR family apoptosis inhibitory protein (NAIP) is highly expressed in M2 macrophages, and cellular IAP 1 (cIAP1) and cIAP2 show opposite expression patterns in M1/M2 polarized macrophages with cIAP1 expressed in M2 and cIAP2 preferentially expressed in M1. Interestingly, IAP antagonists can induce the upregulation of NAIP in M2, downregulation of cIAP1 expression in M1 and M2, and high expression of cIAP2 in M1 macrophages (38).

**Figure 1:**
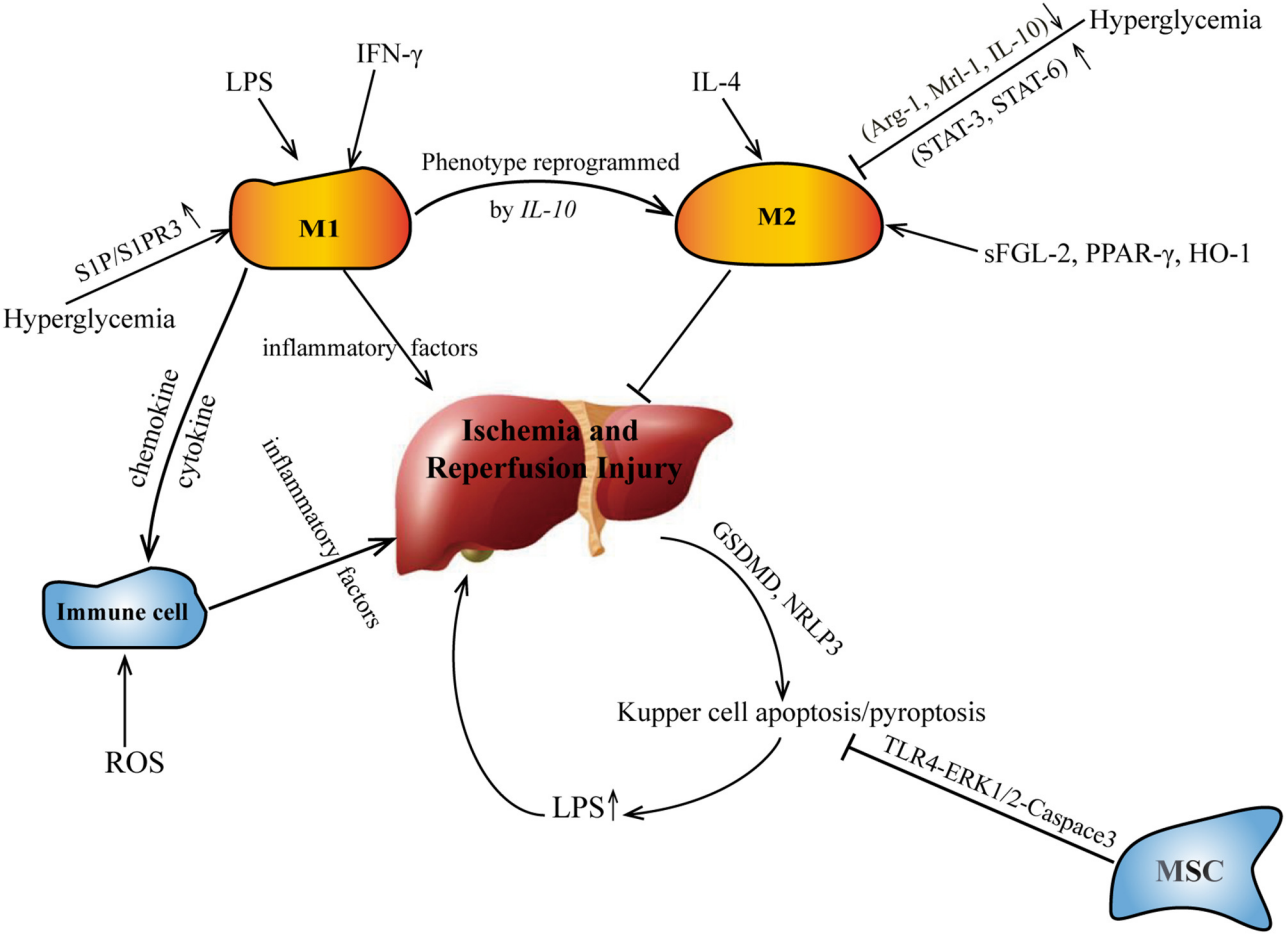
The crosstalk of apoptosis/pyroptosis and polarization of KCs in I/R. IL, interleukin; sFGL, soluble fibrinogen-like protein.

Cell pyrolysis (pyroptosis), also known as cell inflammatory necrosis, is a new type of programmed cell death (39). It is shown that the permeability of the cell membrane changes, resulting in the release of a large amount of cell contents; at the same time, the water outside the cell enters the cell via channels in the cell membrane. This eventually results in the cell lysing to death, triggering a strong inflammation reaction (40). Liver I/R may promote the pyroptosis of KCs mediated by GSDMD and NRLP3 (41). M2-type KCs promote M1-type KC apoptosis through an IL-10-mediated arginase-dependent mechanism (42). The crosstalk of apoptosis/pyroptosis and polarization of KCs in I/R are shown on Figure 1.

## Conclusion and Future Directions

Because I/R during LT can increase the risk of graft dysfunction, transplant rejection, and organ failure, managing I/R remains a major problem in clinical practice. I/R activates KCs, and these activated KCs can be polarized into two subtypes. M1-type KCs play a pro-inflammatory role, and M2-type KCs play an anti-inflammatory role. The effects of KC apoptosis on I/R development are controversial at present. Interestingly, IL-10 plays a role in attenuating liver I/R, likely through regulating the apoptosis of KCs as well as modifying their polarization. However, the mechanism underlying liver I/R is not yet fully understood, highlighting the importance of continued research and clarifying the role of KCs in all factors involved in I/R. Such an understanding will aid in the development of more accurate and complete treatment strategies for LT. Future research on I/R in LT should be aimed at developing new therapeutic interventions, implementing prognostic biomarkers based on KCs, and designing clinical studies. It will be necessary to identify new technologies based on regulating the polarization and apoptosis of KCs to encourage the macrophage population to develop in a direction that results in beneficial rather than harmful inflammatory responses.

## Author Contributions

LY, SH, XM, YZ, YC, and SL contributed to the writing and editing of the manuscript. All authors contributed to the article and approved the submitted version.

## Conflict of Interest

The authors declare that the research was conducted in the absence of any commercial or financial relationships that could be construed as a potential conflict of interest.

## Abbreviations

LT: liver transplantation
I/R: ischemia-reperfusion injury
KC: Kupffer cells
ROS: reactive oxygen species
TNF-α: tumor necrosis factor α
IL: interleukin
DAMPs: Damage-associated molecular patterns
sFGL2: Soluble fibrinogen-like protein 2
DC: dendritic cells.
